# Serum levels of circulating microRNA-107 are elevated in patients with early-stage HCC

**DOI:** 10.1371/journal.pone.0247917

**Published:** 2021-03-12

**Authors:** Sven H. Loosen, Mirco Castoldi, Markus S. Jördens, Sanchary Roy, Mihael Vucur, Jennis Kandler, Linda Hammerich, Raphael Mohr, Frank Tacke, Tom F. Ulmer, Ulf P. Neumann, Tom Luedde, Christoph Roderburg

**Affiliations:** 1 Clinic for Gastroenterology, Hepatology and Infectious Diseases, University Hospital Düsseldorf, Medical Faculty of Heinrich Heine University Düsseldorf, Düsseldorf, Germany; 2 Department of Hepatology and Gastroenterology, Charité University Medicine Berlin, Berlin, Germany; 3 Department of Visceral and Transplantation Surgery, University Hospital RWTH Aachen, Aachen, Germany; University of Navarra School of Medicine and Center for Applied Medical Research (CIMA), SPAIN

## Abstract

**Background:**

Early detection of hepatocellular carcinoma (HCC), the most common primary liver malignancy, is crucial to offer patients a potentially curative treatment strategy such as surgical resection or liver transplantation (LT). However, easily accessible biomarkers facilitating an early diagnosis of HCC as well as a reliable risk prediction are currently missing. The microRNA(miR)-107 has recently been described as a driver of HCC in both murine and human HCC but data on circulating miR-107 in HCC patients are scarce. In the present study, we evaluated a potential diagnostic and/or prognostic role of circulating miR-107 in patients undergoing tumor resection or LT for early-stage HCC.

**Methods:**

The Kmplot bioinformatic tool was used to query publicly available databases (including TCGA, GEO and EGA) in order to analyse the prognostic value of tumoral miR-107 expression in HCC patients (n = 372). Serum levels of miR-107 were measured by qPCR in n = 45 HCC patients undergoing surgical tumor resection (n = 37) or LT (n = 8) as well as n = 18 healthy control samples. Results were correlated with clinical data.

**Results:**

A high tumoral expression of miR-107 was associated with a significantly better overall survival compared to patients with low miR-107 expression levels (HR 0.69, 95% CI 0.48–0.99, p = 0.041). In addition, serum levels of miR-107 were significantly higher in HCC patients when compared to healthy controls. However, miR-107 serum levels in HCC patients were independent of different disease etiology, tumor stage or tumor grading. HCC patients with baseline miR-107 expression levels above a calculated ideal prognostic cut-off value (9.82) showed a clear trend towards an impaired overall survival (p = 0.119).

**Conclusion:**

Tumoral miR-107 expression levels are a potential prognostic marker in early stage HCC. Furthermore, we describe a potential role of circulating miR-107 levels as a diagnostic biomarker in patients with early-stage HCC.

## Introduction

Hepatocellular carcinoma (HCC) is the most frequent primary malignancy of the liver and has risen to become the fifth most common cancer worldwide. Due to the epidemic spread of its most important risk factors such as non-alcoholic steatohepatitis (NASH) or viral hepatitis infection, its incidence is still increasing [1]. Despite intensive research efforts, the prognosis of HCC patients is still poor and, particularly in advanced disease stages, mortality almost equals incidence. Surgical tumor resection and liver transplantation (LT) represent two standard therapies that can potentially provide cure for HCC patients at early stage (Barcelona Clinic Liver Cancer [BCLC] 0 or A [2]). Thus, early diagnosis of HCC is essential to enable a surgical and thus potentially curative treatment option. Nevertheless, many patients are facing early tumor recurrence and five years after tumor resection only about 30% of patients are still free of disease recurrence [3]. In addition, liver surgery is still associated with severe complications [4]. Therefore, in many patients, it often remains unclear whether they will benefit from surgery or not. In this context, different prognostic algorithms (including imaging modalities, laboratory markers and the ECOG performance status) have been proposed to discriminate between patients who particularly benefit from liver resection or LT and should therefore be allocated to a surgical approach and those who do not [5].

Micro(mi)RNAs represent a class of small RNAs that do not withhold information to encode for proteins but regulate the expression of their target mRNAs both at a posttranscriptional and posttranslational level [6]. Just recently, a strong role of different miRNAs in the pathophysiology of HCC has been established [7–9]. In line, we have recently demonstrated that miR-193a-5p in down regulated in both murine and human HCC, leading to a more aggressive course of disease [10]. Interestingly, the intratumoral down-regulation of miR-193a-5p was associated with elevated serum levels of this miRNAs in HCC patients [11]. Alongside miR-193a-5p, our array-based analysis in mice revealed a down-regulation of miR-107 (personal communication with Dr. Castoldi), which has been shown to be involved in the regulation of glucose homeostasis and insulin sensitivity in the liver [12].

In the present study, we hypothesized that circulating miR-107 levels might also be regulated in HCC and could potentially function as a novel biomarker. To investigate this hypothesis, an exploratory analysis was conducted that aimed at evaluating a potential diagnostic or prognostic function of miR-107 in the serum of HCC patients who underwent surgical treatment (resection or LT) for early disease stage at our tertiary referral hospital between 2011 and 2017.

## Patients and methods

### Study design

This exploratory observational cohort study was performed to evaluate a potential role of serum miR-107 levels as a diagnostic and/or prognostic biomarker in n = 45 HCC patients undergoing surgical tumor resection (n = 37) or liver transplantation (n = 8) at University Hospital RWTH Aachen. Patients were admitted to the Department of Visceral and Transplantation Surgery at University Hospital RWTH Aachen for HCC tumor resection or LT and consecutively enrolled by physicians into this study between March 2011 and February 2017. The inclusion criteria were: 1. histologically confirmed HCC (after tumor resection or LT), 2. Age >18 years, and 3. serum sample available before tumor resection/LT. The exclusion criteria were: 1. Presence of a second malignancy, 2. Death <72h following surgery due to surgical complications. Demographic details of the study cohort are summarized in Table 1. The decision for or against tumor resection/LT was based on an interdisciplinary tumor board according to internationally recognized standards (e.g. Milan criteria for LT). Blood samples were collected before surgery, centrifuged for 10 min at 2000 g, and serum samples were then stored at −80°C until use. We included a total of n = 18 healthy, cancer-free blood donors with normal values for blood counts, C-reactive protein, kidney and liver function as a control population. The study protocol was approved by the ethics committee of the University Hospital RWTH Aachen, Germany (EK 206/09) and conducted in accordance with the ethical standards laid down in the Declaration of Helsinki. Written informed consent was obtained from every patient.

**Table 1:** Patient characteristics.

	Study cohort
**HCC patients**	45
Gender [%]:	
male-female	68.9–31.1
Age [years, median and range]	66 [42–82]
BMI [kg/m^2^, median and range]	26.57 [17.67–39.18]
Cirrhosis [%]	
Yes	91.1%
No	8.9%
Surgical treatment [%]:	
Tumor resection	82.2
Liver transplantation	17.8
HCC etiology:	
Hepatitis B	8.9
Hepatitis C	22.2
NASH	15.6
Alcoholic	11.1
Others	42.2
T-stage [%]:	
T1	23.5
T2	47.1
T3/4	29.4
Tumor grading [%]:	
G1	9.4
G2	71.9
G3	18.8
Resection status [%]:	
R0	87.1
R1	12.9
Tumor size [cm, median and range]:	4.85 [1.0–24.0]
ECOG PS [%]:	
ECOG 0	53.3
ECOG 1	42.2
ECOG 2	2.2
ECOG 3	2.2
Deceased during follow-up [%]:	
Yes—No	71.1–28.9

HCC: hepatocellular carcinoma, BMI: body mass index, NASH: non-alcoholic steatohepatitis, ECOG PS: „Eastern Cooperative Oncology Group”performance status.

### MiRNA isolation from serum

300μl serum was spiked with *miScript miRNA mimic SV40* (Qiagen, Germany) for sample normalization. 600μl *peqGOLD TriFast™* (VWR) and 150μl chloroform were added to the sample and mixed vigorously for 15 sec followed by an incubation at room temperature for 10 min. Samples were centrifuged for 15 min at 12,000g until complete phase separation. The aqueous phase, containing total RNA, was precipitated with 375μl 100% isopropanol and 1.5μl glycogen (Fermentas, St. Leonroth, Germany) overnight at -20°C. After centrifugation at 4°C for 30 min (12,000 g) the pellets were washed once with 70% ethanol and centrifugation at 12000 g, 5 min and 4°C. Precipitated RNA was resuspended in 30μl RNase free water.

### Quantitative reverse transcriptase PCR (qPCR)

Total RNA was used to synthesize cDNA utilizing miScript Reverse Transcriptase Kit (Qiagen) according to the manufacturer’s protocol, and was resuspended in suitable amounts of H_2_O. cDNA samples (2 μl) were used for quantitative PCR in a total volume of 25 μl using the miScript SYBR Green PCR Kit (Qiagen) and miRNA specific primers (Qiagen) on a PCR machine (Applied Biosystems 7300 Sequence Detection System, Applied Biosystems, Foster City, CA). Data using the 2^-ΔΔCT^ method were presented as relative gene expression. Data were generated and analyzed using the SDS 2.3 and RQ manager 1.2 software packages (Applied Biosystems).

### Statistical analysis

All statistical analyses were performed using SPSS 23 (SPSS, Chicago, IL, USA) and RStudio (v1.2.5033, RStudio, Inc., Boston, MA, USA) [8]. A p-value of < 0.05 was considered statistically significant (* p < 0.05; ** p < 0.01; *** p < 0.001). Shapiro-Wilk-Test was performed to test for normal distribution. Non-parametric data were compared with the Mann-Whitney-U-Test or the Kruskal-Wallis-Test in case of multiple group comparisons. Correlation analyses were performed using the Spearman’s correlation coefficient. ROC curves were generated by plotting the sensitivity against 1-specificity. Optimal cut-off values for ROC curves were calculated with the Youden-Index method (YI = sensitivity + specificity—1). Kaplan-Meier curves display the impact of miR-107 expression levels on overall survival (OS). The Log-rank test was used to test for statistical differences between subgroups. The ideal cut-off value for the identification of patients with an impaired OS was calculated using a univariate binary cox proportional hazard model and testing for the minimum p-value in RStudio.

## Results

### Tumoral miR-107 expression levels are a prognostic marker for overall survival in HCC patients

**Fig 1:**
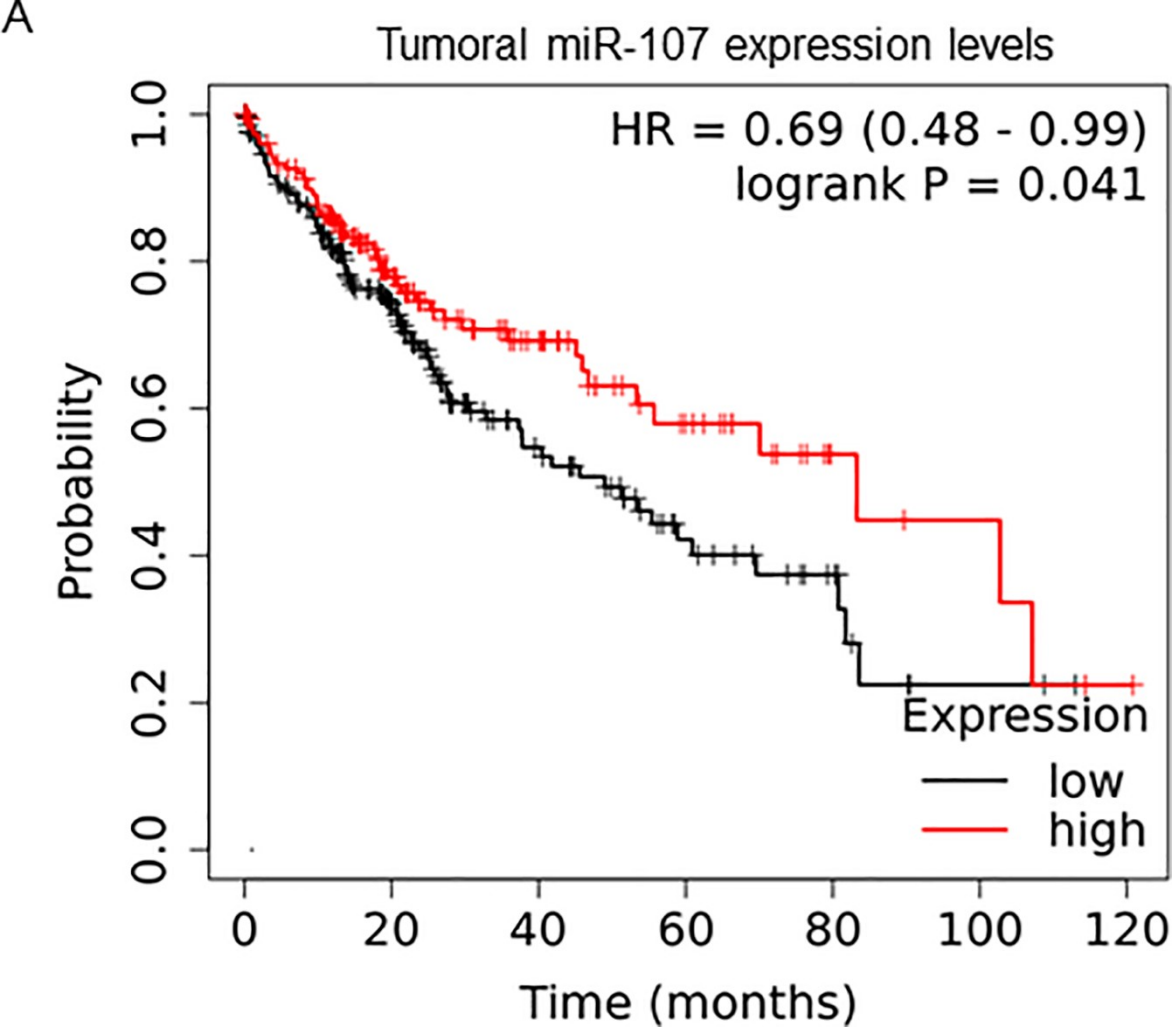
MiRNA-107 expression levels in HCC tissue represent a prognostic marker. Kmplot was used to analyze the prognostic value of miR-107 in HCC in NGS data from TCGA, GEO, and EGA (n = 372 patients). Patients were automatically split by Kmplot in two groups with low (n = 214) and high (n = 158) levels for miR-107 expression (expression range, 70–989, cut off expression: 270,22). Overall survival of HCC patients with high tumoral miR-107 expression levels is significantly higher compared to patients with low miR-107 expression levels (HR 0.69, 95% CI 0.48–0.99, p = 0.41).

The Kmplot bioinformatic tool was used to query publicly available databases (including TCGA, GEO and EGA), with available RNA-seq data of HCC patients [13]. Specifically, Kmplot was used to analyze the prognostic value of miR-107 in n = 372 HCC patients. Kmplot automatically assigned patients to two groups with low (n = 214) and high (n = 158) miR-107 expression (cut off 270.22)Patients with high tumoral expression levels of miR-107 had a significantly better overall survival compared to patients with low levels of miR-107 expression in tumor tissue (HR 0.69, 95% CI 0.48–0.99, p = 0.041). Median overall survival was 83.24 months in the miR-107-high group and 48.99 months in the miR-107-low group (Fig 1).

### Baseline characteristics of HCC cohort

Based on the tumoral expression data suggesting a crucial role of miR-107 in HCC, we next aimed at measuring levels of circulating miR-107 in patients with early-stage HCC, representing the patient group being at highest need for the development of markers suitable for early detection of cancer. Between 2011 and 2017, we enclosed a total of n = 45 patients who presented with HCC and were allocated to either tumor resection (n = 37) or liver transplantation (LT, n = 8) [11]. The median age of the study population was 66 years (range: 42–82 years). 68.9% of patients were male and 31.1% female. 91.1% of patients were diagnosed with liver cirrhosis, 8.9% had no cirrhosis. The underlying disease etiology was distributed as follows: 8.9% hepatitis B, 22.2% hepatitis C, 15.6% NASH, 11.1% alcoholic liver disease and 42.2% others. The median HCC tumor size was 4.85 cm. Table 1 provides a detailed characterization of the study population.

### Levels of circulating miR-107 are upregulated in HCC patients

**Fig 2:**
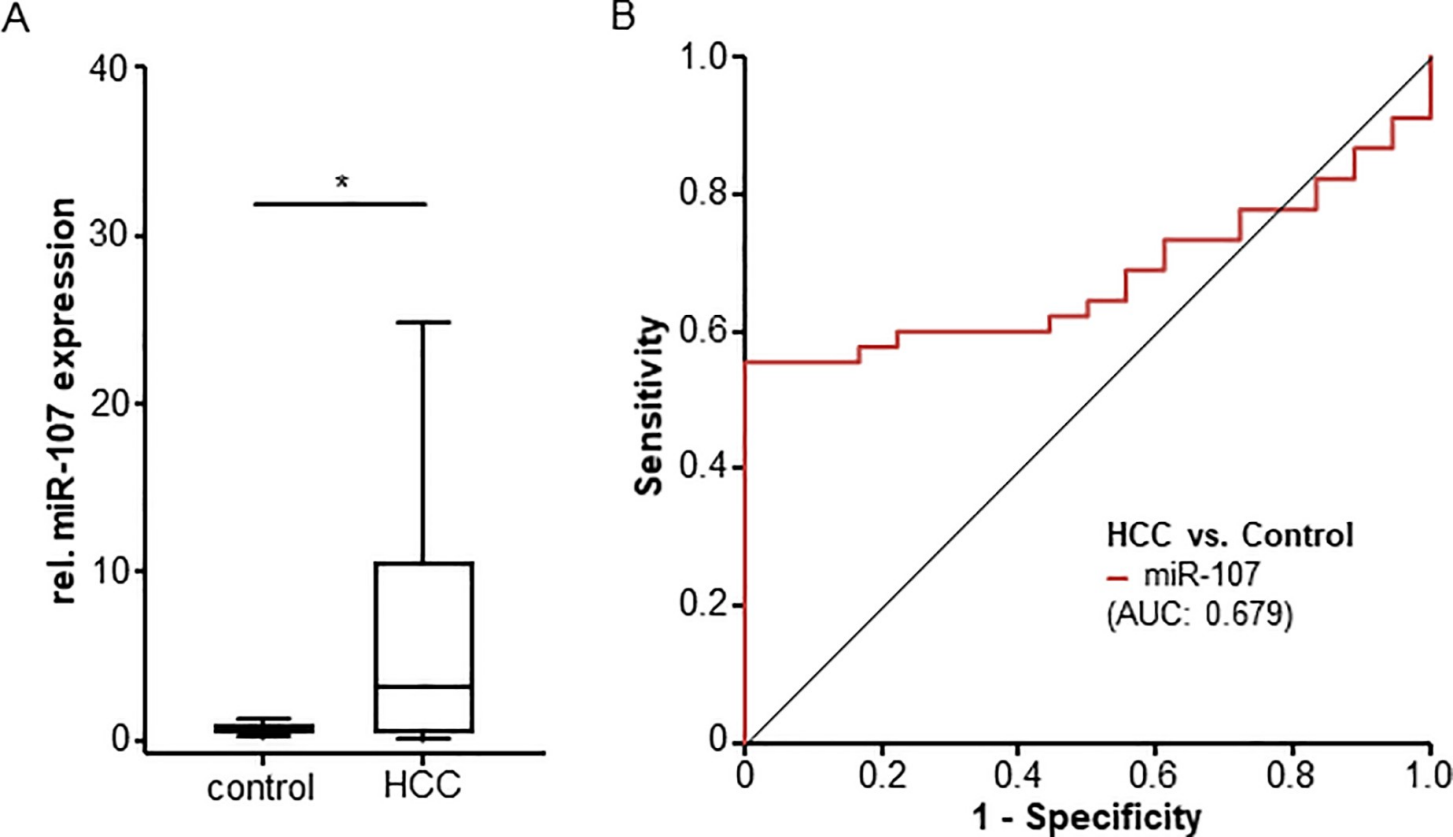
MiRNA-107 levels are upregulated in HCC patients. (A) Circulating miR-107 levels are significantly higher in HCC patients compared to healthy controls. (B) Circulating miR-107 levels show an AUC value of 0.679 regarding the discrimination between HCC and healthy controls.

In a first step, we compared circulating levels of miR-107 between HCC patients and healthy controls (Fig 2A). Notably, miR-107 serum levels were significantly higher in the HCC patient group, demonstrating a 4.3-fold induction when compared to healthy control samples (median HCC: 3.12, median healthy controls: 0.725, Table 2). Despite this strong elevation, ROC curve analysis revealed an only modest diagnostic potential of elevated serum miR-107 expression levels, showing an AUC value of 0.679 for the discrimination between individuals with HCC and controls (Fig 2B). At the optimal diagnostic cut-off value of 2.63, miR-107 expression levels showed a sensitivity and specificity of 55.6 and 100% regarding the diagnosis of HCC.

**Table 2:** Serum levels of various laboratory markers.

	HCC patients	Healthy controls
	median [range]	median [range]
rel. miR-107 expression	3.12 [0.03–79.04]	0.725 [0.16–2.32]
Leucocyte count [cells/nl]	6.6 [3.7–16.5]	-
Haemoglobin [g/l]	13.2 [8.5–16.5]	-
Platelets [cells/nl]	174.0 [19.0–754.0]	-
Sodium [mmol/l]	139.0 [126.0–144.0]	-
Potassium [mmol/l]	4.4 [3.3–5.9]	-
Bilirubin [mg/dl]	0.71 [0.26–3.60]	-
AST [U/l]	48.0 [19.0–439.0]	-
ALT [U/l]	31.0 [7.0–168.0]	-
GGT [U/l]	145.0 [27.0–794.0]	-
ALP [U/l]	108.5 [61.0–371.0]	-
Creatinine [mg/dl]	0.93 [0.46–2.18]	-
CRP [mg/l]	6.70 [0–162.6]	-
AFP [μg/l]	17.6 [2.0–55,368.0]	-

miR: microRNA, AST: aspartate transaminase, ALT: alanine transaminase, GGT: γ-Glutamyl transpeptidase, ALP: alkaline phosphatase, CRP: C-reactive protein, AFP: alpha-fetoprotein.

### Serum miR-107 levels do not correlate with patients’ characteristics

**Fig 3:**
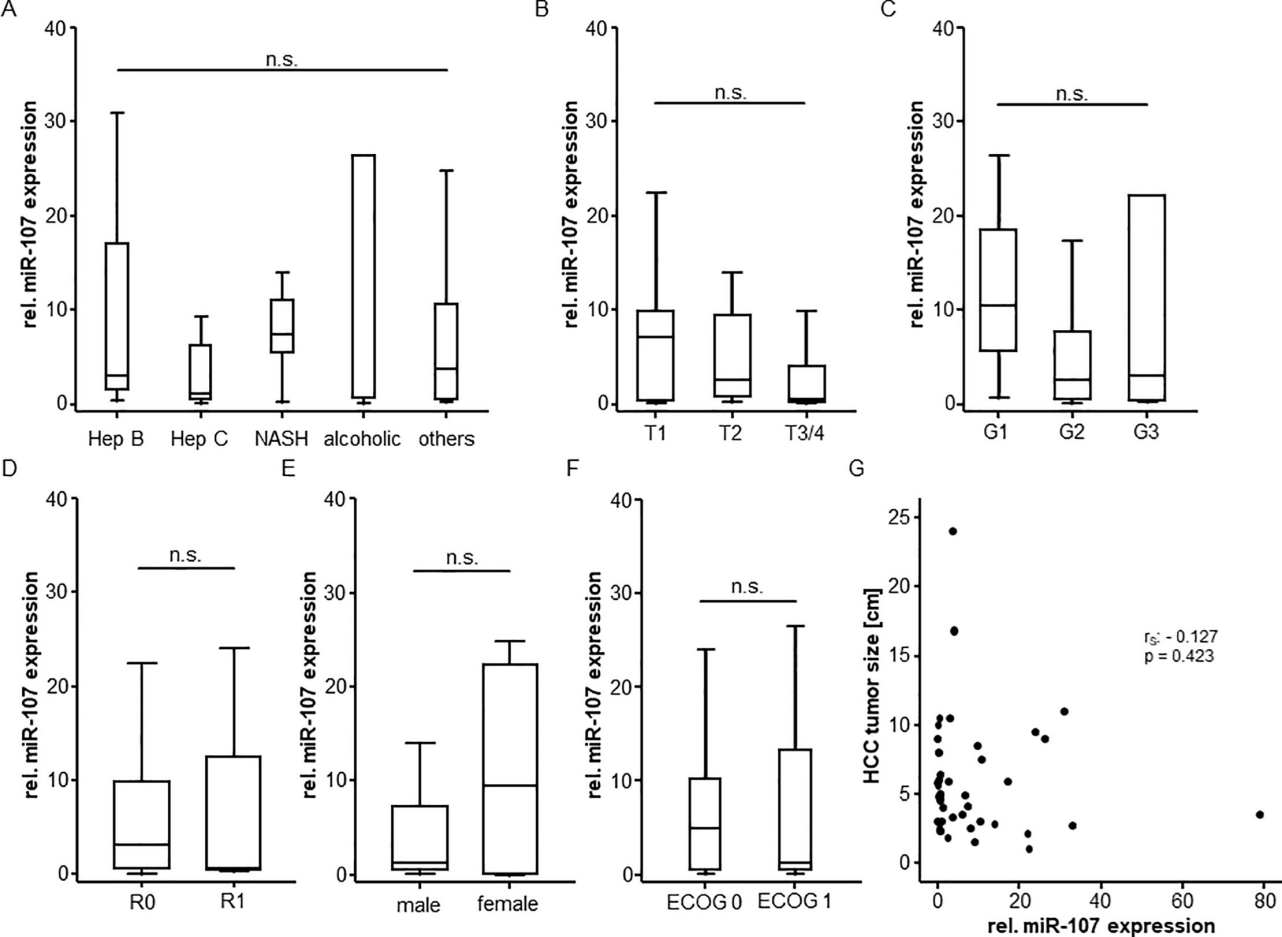
Circulating miR-107 levels in different HCC subgroups. Serum miR-107 levels do not significantly differ between patients with different liver disease etiology (A), tumor stage (B), tumor grading (C), surgical resection status (D) as well as between male and female patients (E) or patients with an unimpaired/impaired ECOG performance status (F). MiR-107 serum levels do not correlate with the size of HCC (G).

In order to understand the mechanism regulating miR-107 serum concentrations in patients with early-stage HCC, we next compared concentrations between different subgroups of patients. However, serum expression levels of miR-107 were independent of the underlying liver disease since patients with hepatitis B or C virus infection displayed almost identical levels of miR-107 compared to those with NASH or those with alcoholic hepatitis (Fig 3A). Moreover, neither the specific tumor stage according to the TNM classification (Fig 3B), nor the tumor grading (Fig 3C) had an influence on circulating miR-107 levels. Of note, these findings were supported by the fact that the resection status (resected patients only, Fig 3D), the patient’s sex (Fig 3E), the ECOG performance status (Fig 3F) and the tumor size (Fig 3G) did not affect serum levels of miR-107.

To get a deeper insight into the regulation of circulating miR-107, we next performed correlation analyses between miR-107 concentrations and laboratory parameters routinely used in patients with HCC and/ or liver cirrhosis. However, we did not observe a significant correlation between serum miR-107 levels and parameters of liver dysfunction (bilirubin, AST, ALT, GGT, ALP), systemic inflammation (leucocyte count and CRP), renal dysfunction (creatinine), established HCC tumor markers (AFP) as well as hemoglobin and the platelet count (Table 3).

**Table 3:** Correlation analysis between miR-107 expression levels and various laboratory markers.

Parameter	Correlation coefficient (r_S_)	p-value
AST	0.017	0.911
ALT	-0.146	0.459
Bilirubin	-0.178	0.243
GGT	0.165	0.295
ALP	0.055	0.738
AFP	-0.135	0.510
Sodium	0.002	0.991
Potassium	0.089	0.561
Calcium	-0.056	0.717
Hemoglobin	-0.109	0.474
Leucocytes	-0.120	0.341
Platelets	0.108	0.479
CRP	0.255	0.103
Creatine	-0.085	0.579

AST: aspartate transaminase, ALT: alanine transaminase, GGT: γ-Glutamyl transpeptidase, ALP: alkaline phosphatase, AFP: alpha-fetoprotein, CRP: C-reactive protein.

### Serum miR-107 levels are unsuitable to predict outcome in resectable HCC

**Fig 4:**
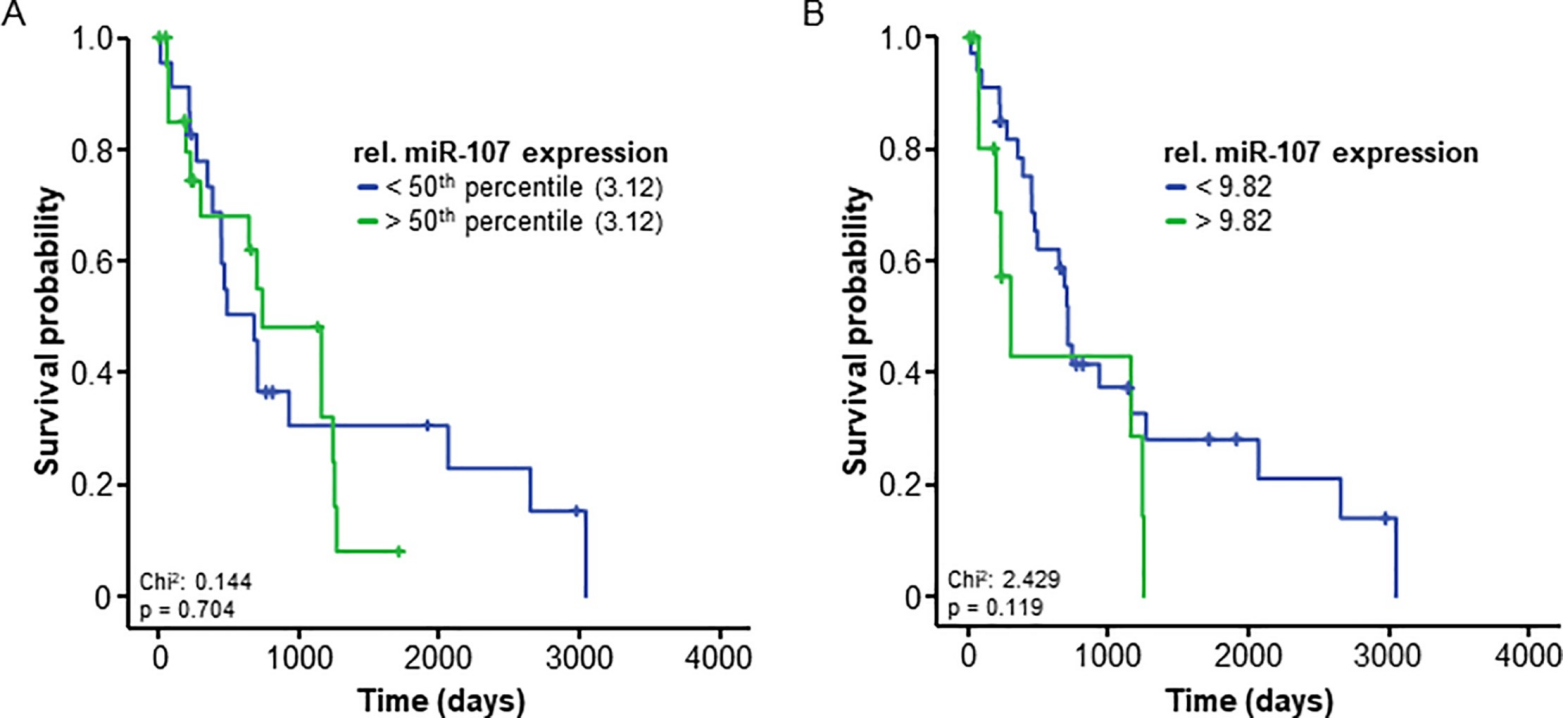
Serum levels of circulating miR-107 are unsuitable to predict outcome in early-stage HCC patients. (A) HCC patients with miR-107 serum levels above or below the 50^th^ percentile (3.12) have a comparable overall survival. (B) HCC patients with miR-107 serum levels above the ideal prognostic cut-off value (9.82) show a non-significant trend (p = 0.119) towards an impaired survival.

Different circulating miRNAs recently turned out as prognostic markers for HCC [14]. Based on these findings as well as on our own data indicating a role of miR-107 in the pathophysiology of HCC (Fig 1), we hypothesized that expression levels of circulating miR-107 might also be indicative for the patient’s outcome following surgical therapy. We used Kaplan-Meier curve analysis to compare the overall survival (OS) of patients with elevated levels of circulating miR-107 (above the median of all patients) and those with low miR-107 levels. Interestingly, both groups demonstrated a comparable OS (Fig 4A). As the median might not be the optimal cut-off for the discrimination between patients with a favorable/unfavorable prognosis, we aimed at establishing a statistically optimal prognostic cut-off value by using a univariate binary cox proportional hazard model and testing for the minimum p-value as recently described [11]. By applying this cut-off value (9.82 relative units), we observed a strong trend towards an impaired survival in patients with miR-107 levels > 9.82, however the difference failed statistical significance (p = 0.119). Notably, median OS was only 300 days for the subgroup of patients showing high miR-107 concentrations compared to 707 days in those patients with low miR-107 levels (Fig 4B). In univariate Cox-regression analysis, miR-107 expression levels showed a HR or 1.023 (0.995–1.051, p = 0.105) for the prediction of OS.

## Discussion

In the present study, we demonstrate that tumoral miR-107 expression levels are a predictive marker for overall survival in unselected HCC patients. Increased expression levels indicate a significantly improved prognosis, while low expression levels in the tumor tissue are associated with a statistically significant reduced overall survival compared. Tumoral miR-107 expression in human seems to differ from mice (personal communication with Dr. Castoldi). Differences in tumoral miR-107 expression could be explained by the different role of the transcription factor PPARalpha in mice and human, which is suggested to play a role in the miR-107 transcription [15]. While PPARalpha activators in mice seem to promote hepatocarcinogenesis this seems to be not the case in human [16, 17].

In line with our data, it was recently shown that increased serum levels of miR-107 indicate response to TACE treatment [18]. In this study, several miRNAs were investigated for their potential to predict outcome to TACE treatment. Beside the prognostic value of expression levels in the tumor tissue itself, miR-107 serum levels were described as a potential prognostic marker for therapy response. Furthermore, as well consistent with our data, increased miR-107 serum levels were detected in patients with resectable but also advanced HCC in a Chinese cohort [19]. Especially in combination with two other miRNA (miR-92a-3p and miR-3126-5p), a good prognostic value for the diagnosis of HCC of all stages was found. Therefore, we aimed at investigating whether serum levels of miR-107 might be useful as a diagnostic or prognostic marker in early-stage HCC Caucasian patients. In our cohort, miR-107 serum levels were significantly higher in patients with resectable HCC compared to healthy controls. The elevation was independent of the underlying liver disease, making miR-107 a potential valuable marker for general HCC screening and diagnosis in early HCC stage. Also, the tumor grading had no significant influence on the level increase, suggesting that miR-107 might be suitable to detect even highly differentiated tumors, representing a major challenging in screening of patients with liver cirrhosis. We next analyzed whether, along with its role as a diagnostic marker in HCC, miR-107 levels could also be used as a prognostic marker in HCC. However, we found no significant difference in overall survival between patients with low or high miR-107 serum levels in our cohort. However, in our study only patients with potentially curable tumor disease were included. This could be an explanation for the divergent results in serum compared to the tissue tests. Further investigations with HCC patients in non-curative stages and locally advanced or metastatic disease are necessary to clarify this question. Importantly, expression analysis of miR-107 in tumor tissue and serum were performed in two different cohorts of HCC patients and we were thus unable to evaluate a direct correlation of miR-107 expression levels.

Early detection of HCC in patients with liver cirrhosis is important to offer a surgical and thus potentially curative treatment to these patients. Although there have been controversial studies on screening for HCC in recent years [20], screening examinations for HCC are still firmly established in the major guidelines of the various professional societies. For this purpose, 6-month sonography of the liver is of particular importance. However, the additional benefit of biomarker testing is controversially discussed. The obligatory additional AFP measurement was recently no longer recommended in some major HCC guidelines [5], as the additional benefit of AFP measurement was evaluated differently in several studies. However, a recent meta-analysis of HCC surveillance, which included a total of 32 studies involving 13,367 patients, suggests an additional AFP measurement to the ultrasound examination. Ultrasound alone had a lower sensitivity of 45% for HCC detection than the combination with AFP determination at 63% (relative risk 0.88; 95% CI 0.83–0.93 for all stages, early-stage RR 0.81; 95% CI 0.71–0.93) [21]. These uncertainties in HCC screening have therefore fueled the search for new and better biomarkers or the combination of these to optimize HCC monitoring. For example, the GALAD-Score was established, which determines the HCC risk by patient age, gender and the biomarkers -fetoprotein (AFP), AFP isoform L3 (AFP-L3) and des-gamma-carboxy prothrombin (DCP). The overall sensitivity and specificity of this test procedure was significantly better than the AFP determination alone [22]. One might hypothesize that including additional markers, such as miRNA might further increase the value of this test. In particular as several miRNA were proven suitability for HCC screening [23].

In recent years, the potential of miRNA for diagnostics but also for estimating prognosis in HCC has been discovered. In particular, the focus has been on alterations of the expression of certain miRNAs in the tumor tissue itself. High expression of miR-32 in HCC tissue was identified as a negative prognostic marker. Patients with high expression of miR-32 in tumor tissue had significantly worse PFS and OS comparted to patients with low expression levels [24]. A similar situation has been shown for miR-221. Again, increased expression levels in the tumor seem to be associated with a poorer prognosis [25]. In contrast, downregulation of miR-33a in tumor tissue seems to be a negative prognostic factor for HCC [26]. Different miRNAs were also investigated as diagnostic biomarkers for HCC in the screening. In patients with chronic hepatitis B or C, microRNA-139 was found to be decreased in HCC [27, 28]. There are different results for mircoRNA-182. On the one hand an increased risk of HCC in case of upregulation was described, on the other hand a significant downregulation of the same miRNA in HCC patients with chronic hepatitis C [29, 30]. Other microRNAs such as miR-150, miR-331-3p or miR-193 were suggested being diagnostic markers or markers for the prediction of disease progression [11, 27, 29]. It should be noted that miRNA expression in tumor tissue is often used as diagnostic or prognostic marker. This is certainly more difficult to practice in clinical routine than the determination of serum levels. This could be an advantage of miR-107 determination in serum. With miR-107, we investigated a new candidate with similar sensitivity than other biomarkers but very high specificity for HCC screening especially in patients with still resectable HCC. It is important to investigate the value of this candidate as the only screening parameter or in combination with other miRNAs or biomarkers. This could lead to a new screening score for HCC in cirrhotic or non-cirrhotic patients with high-risk constellation for the HCC development. Furthermore, miR-107 is already elevated in early-stage HCC, making it valuable for screening. For its prognostic potential in HCC, it might share the same problem than other miRNAs. As for miR-32 or miR-221, at present also for miR-107 tumor tissue is needed to be able to use it as a prognostic marker. Serum levels–at least in early-stage HCC–seem to not mirror the prognosis of the disease in a sufficient manner.

The common limitation of all novel biomarkers—including miR-107—is certainly the lack of broad availability and sometimes complicated testing. There is also no meaningful data on cost efficiency. Further investigations are therefore necessary for a general recommendation for screening, diagnostics or prognosis. As noted, further studies with miR-107 serum levels in later stage HCC patients are important for the evaluation of this marker as a diagnostic and maybe also prognostic tool.
